# Identification of a possible proteomic biomarker in Parkinson’s disease: discovery and replication in blood, brain and cerebrospinal fluid

**DOI:** 10.1093/braincomms/fcac343

**Published:** 2022-12-28

**Authors:** Laura Winchester, Imelda Barber, Michael Lawton, Jessica Ash, Benjamine Liu, Samuel Evetts, Lucinda Hopkins-Jones, Suppalak Lewis, Catherine Bresner, Ana Belen Malpartida, Nigel Williams, Steve Gentlemen, Richard Wade-Martins, Brent Ryan, Alejo Holgado-Nevado, Michele Hu, Yoav Ben-Shlomo, Donald Grosset, Simon Lovestone

**Affiliations:** Department of Psychiatry, University of Oxford, Oxford OX3 7JX, UK; Department of Psychiatry, University of Oxford, Oxford OX3 7JX, UK; Population Health Sciences, Bristol Medical School, University of Bristol, Bristol, UK; Department of Psychiatry, University of Oxford, Oxford OX3 7JX, UK; Department of Psychiatry, University of Oxford, Oxford OX3 7JX, UK; Oxford Parkinson's Disease Centre and Division of Neurology, Nuffield Department of Clinical Neurosciences, University of Oxford, Oxford, UK; Division of Psychological Medicine and Clinical Neurosciences, MRC Centre for Neuropsychiatric Genetics and Genomics, Cardiff University, Cardiff, Wales, UK; Division of Psychological Medicine and Clinical Neurosciences, MRC Centre for Neuropsychiatric Genetics and Genomics, Cardiff University, Cardiff, Wales, UK; Division of Psychological Medicine and Clinical Neurosciences, MRC Centre for Neuropsychiatric Genetics and Genomics, Cardiff University, Cardiff, Wales, UK; Oxford Parkinson's Disease Centre, Kavli Institute for Nanoscience Discovery, Department of Physiology, Anatomy and Genetics, University of Oxford, Oxford, UK; Division of Psychological Medicine and Clinical Neurosciences, MRC Centre for Neuropsychiatric Genetics and Genomics, Cardiff University, Cardiff, Wales, UK; Department of Brain Sciences, Imperial College London, London, UK; Oxford Parkinson's Disease Centre, Kavli Institute for Nanoscience Discovery, Department of Physiology, Anatomy and Genetics, University of Oxford, Oxford, UK; Oxford Parkinson's Disease Centre, Kavli Institute for Nanoscience Discovery, Department of Physiology, Anatomy and Genetics, University of Oxford, Oxford, UK; Department of Psychiatry, University of Oxford, Oxford OX3 7JX, UK; Oxford Parkinson's Disease Centre and Division of Neurology, Nuffield Department of Clinical Neurosciences, University of Oxford, Oxford, UK; Population Health Sciences, Bristol Medical School, University of Bristol, Bristol, UK; Institute of Neuroscience and Psychology, University of Glasgow, Glasgow, UK; Department of Psychiatry, University of Oxford, Oxford OX3 7JX, UK

**Keywords:** Parkinson’s disease, proteomics, biomarker

## Abstract

Biomarkers to aid diagnosis and delineate the progression of Parkinson’s disease are vital for targeting treatment in the early phases of the disease. Here, we aim to discover a multi-protein panel representative of Parkinson’s and make mechanistic inferences from protein expression profiles within the broader objective of finding novel biomarkers. We used aptamer-based technology (SomaLogic®) to measure proteins in 1599 serum samples, 85 cerebrospinal fluid samples and 37 brain tissue samples collected from two observational longitudinal cohorts (the Oxford Parkinson’s Disease Centre and Tracking Parkinson’s) and the Parkinson’s Disease Brain Bank, respectively. Random forest machine learning was performed to discover new proteins related to disease status and generate multi-protein expression signatures with potential novel biomarkers. Differential regulation analysis and pathway analysis were performed to identify functional and mechanistic disease associations. The most consistent diagnostic classifier signature was tested across modalities [cerebrospinal fluid (area under curve) = 0.74, *P* = 0.0009; brain area under curve = 0.75, *P* = 0.006; serum area under curve = 0.66, *P* = 0.0002]. Focusing on serum samples and using only those with severe disease compared with controls increased the area under curve to 0.72 (*P* = 1.0 × 10^−4^). In the validation data set, we showed that the same classifiers were significantly related to disease status (*P* < 0.001). Differential expression analysis and weighted gene correlation network analysis highlighted key proteins and pathways with known relationships to Parkinson’s. Proteins from the complement and coagulation cascades suggest a disease relationship to immune response. The combined analytical approaches in a relatively large number of samples, across tissue types, with replication and validation, provide mechanistic insights into the disease as well as nominate a protein signature classifier that deserves further biomarker evaluation.

## Teaser

Winchester *et al*. used a Parkinson’s disease protein study with combined multiple cohorts and different measures (blood, brain and cerebrospinal fluid) to find a multi-protein panel representative of Parkinson’s and understand more about disease mechanisms from protein expression profiles.

## Graphical Abstract

**Graphical abstract:**
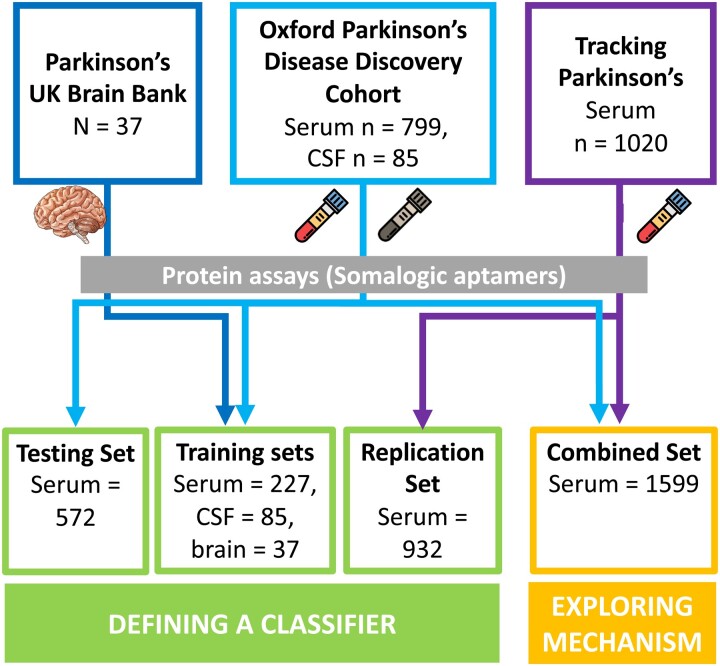

## Introduction

Parkinson’s disease is a complex neurological disorder resulting in disabling motor and non-motor deficits, often accompanied by cognitive impairment, as a consequence of the loss of dopaminergic neurons within the substantia nigra pars compacta and ventral tegmental area.^1^ Given the prevalence of over 350 per 100 000 people globally which increases with age,^2^ Parkinson’s disease represents a heavy burden not only to patients but to families, careers and society. It has become increasingly apparent that there is a prolonged prodromal phase of Parkinson’s disease,^3^ similar to other neurodegenerative conditions such as Alzheimer’s disease.^4^ A biomarker(s) for use in selection and stratification in clinical trials and early therapeutic intervention has become an increasingly pressing objective for research as we seek disease-modifying therapies. In Alzheimer’s disease, fluid biomarkers of pathological processes have been identified in both cerebrospinal fluid (CSF) and blood and are increasingly used in clinical trials.^5^ In Parkinson’s disease, reliable biomarkers for use in clinical settings that recapitulate underlying pathophysiology are rare, making the search for candidate protein markers more urgent.^6^ Basso *et al.*^7^ described 44 proteins that supported the role of oxidative stress. Subsequently, Chen-Plotkin *et al.*^8^ used immunoassays of 151 proteins in plasma and found epidermal growth factor to be associated with cognitive decline in Parkinson’s disease. In addition to targeted biomarker testing, biomarker identification has been broadened to include unsupervised discovery approaches. Chen-Plotkin used the SomaLogic® aptamer capture technology to identify a putative Parkinson’s disease-related signature, finding four new markers of Parkinson’s disease that were able to differentiate Parkinson’s disease from amyotrophic lateral sclerosis patients.^9^ Serum neurofilament light chain levels have been shown to be raised in Parkinson’s disease compared with controls, with longitudinal data showing positive associations with motor scores.^10^ These results and those from other studies suggest that there might be a signature of disease in peripheral fluids such as blood, with the potential for use as a biomarker.^6,11^

We sought to build on past studies and learn from similar approaches in other neurodegenerative diseases^12–14^ to identify protein-based biomarkers from peripheral fluids in Parkinson’s disease in larger cohorts. We used high dimensionality proteomics technology and slow-off-rate modified aptamers (SOMAmer® assay, SomaLogic®) based on aptamer capture assays of between 1000 and 4000 proteins. Here, we describe a dual approach to the study analysis; a machine-learning (ML) approach to discover a reproducible multi-protein signature representative of Parkinson’s disease and an in-depth analysis of proteomic function to provide mechanistic disease insights.

## Materials and methods

### Study design and participants

**Figure 1:**
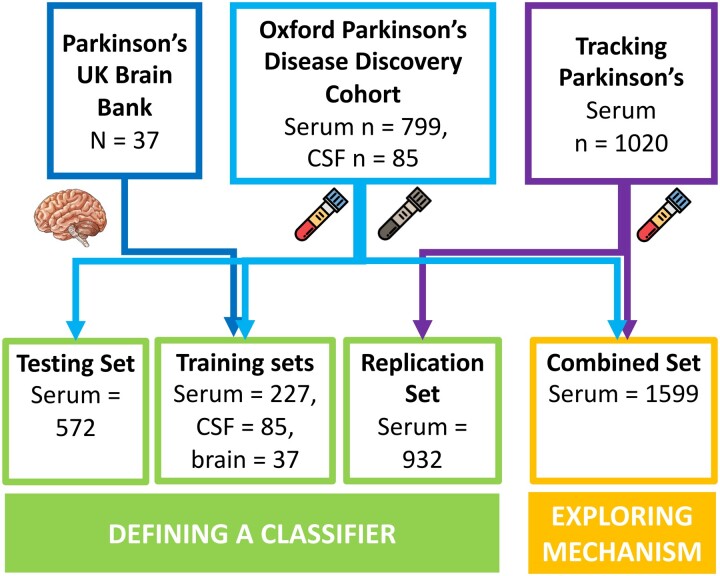
**The MAP2PD—multiple cohort study design.** The two approaches were used to analyse the samples from the MAP2PD. To define a classifier, sample sets from all three cohorts were used. Differential regulation and enrichment analysis (exploring mechanisms) were performed on serum samples only. Brain, post-mortem brain tissue; Serum, blood-derived serum sample.

Parkinson’s disease cases and control samples for this study were obtained from three cohorts: the Oxford Parkinson’s Disease Centre (OPDC) discovery cohort,^15^ the tracking Parkinson’s (TP) cohort (Parkinson’s Repository of Biosamples and Networked Data sets—PRoBaND)^16^ and the Parkinson’s UK Brain Bank (PUKBB).^17^ Samples were organized and tested by the Mapping Proteomics to Parkinson’s disease-UK collaboration with a multi-stage design including blood, brain and CSF samples (Fig. 1).

### Oxford Parkinson’s Disease Centre cohort

The OPDC discovery cohort was collected from 11 hospitals covering a UK Thames Valley population of 2.1 M. Cases were selected using clinical data permitting endophenotype analysis by the severity of the motor phenotype [both measured using the Movement Disorder Society—Unified Parkinson's Disease Rating Scale (MDS-UPDRS) scores] and by cognitive impairment [measured using montreal cognitive assessment (MoCA)]. Individuals with an alternative diagnosis or with a clinical impression of Parkinson’s disease probability <90% at their latest visit were excluded. Levodopa equivalent daily dose (LEDD) was recorded at the time of sampling. All individuals with a baseline disease duration of >3.5 years were excluded. For the initial sampling, using the OPDC-Serum-1K, disease-free controls were matched by sex and age, where age matching was within ±2.5 years. Individuals with common LRRK2 or GBA mutations were excluded. The OPDC-Serum-1K set contained 144 samples, including 65 controls. CSF proteins were measured in 85 samples. The OPDC-Serum-4K set contained 400 cases and 172 controls, here the larger sample set incorporated all available controls and mutation exclusion filters were not required.

### Tracking Parkinson’s cohort

The TP or PRoBaND cohort is a prospective study spanning 72 UK locations. The two sets of samples were selected from this cohort. An initial set (TP-Serum-1K) and a furthermore comprehensive set (TP-Serum-4K). The TP-Serum-1K set contained 80 cases and was combined with the OPDC-serum-1K for analysis. Age matching was within 2.5 years. The TP-Serum-4K sample set (*n* = 1020) includes a broader range of endophenotypes from the clinical measures. All individuals with a baseline disease duration of >3.5 years were excluded. Individuals with an alternative diagnosis or with a clinical impression of Parkinson’s disease probability <90% at their latest visit were excluded. In both sample sets, LEDD was recorded at the time of sampling.

### Parkinson’s UK Brain Bank

The PUKBB is part of the Multiple Sclerosis Society and the Parkinson’s Tissue Bank based at the Imperial College London. About 37 brain (anterior cingulate cortex) samples were used for the brain test set, all originating from the PUKBB cohort. Twenty-four brain samples were from Parkinson’s disease donors and 13 brain samples were from control donors. Samples were selected, which had a post-mortem interval of <26 h and RNA integrity of >6.0.

### Ethics/patient consent

The OPDC study was undertaken with the understanding and written consent of each subject, with the approval of the National Research Ethics Service Committee South Central—Oxford A (Ref: 16/SC/0108); Oxford C (Ref: 15/SC/0117) and the Berkshire Research Ethics Committee (Ref: 10/H0505/71). This was in compliance with national legislation and the Declaration of Helsinki. The TP study is carried out in accordance with the Declaration of Helsinki. Patient consent was obtained and ethics approval was made by the West of Scotland Ethics Committee. The PUKBB is part of the Multiple Sclerosis Society and Parkinson’s Tissue Bank at the Imperial College, London. It has been approved as a research tissue bank by the Wales Research Ethics Committee (Ref: 08/mre09/31 + 5).

### Classifying disease severity in the cohorts

Case samples were split into ‘mild’ and ‘severe’ phenotypes, defined by cognitive and motor symptoms, in order to make inferences about changes in disease pathology during disease progression. Initially, for the OPDC-Serum-1K subset, samples were selected using the bottom 15% (mild) and the top 10% (severe), with a few intermediate cases in between. For the final phase of the analysis, an equivalent approach was developed to generate a severity score per patient (Supplementary Methods). This score was used to sort samples by being above or below the median. The severity score was used as a continuous measure in the larger validation set.

### Protein measurements

All samples analysed in this study had protein levels measured using a SOMAmer®–based capture array in five batches using this evolving array (‘SOMAscan®’ assay from SomaLogic®, Inc. Boulder, CO).^18^ Over the course of the study, this platform was upgraded—namely, OPDC and brain samples were run on the Version 2 panel, which includes 1129 proteins, TP and CSF samples were run on an updated panel (Version 3), with a total of 4006 proteins including those on Version 2. Protein concentration measurements were derived from relative fluorescent units, which had undergone Somalogic® proprietary experimental normalization to standardize noise between sample plates.

### Quality control and data pre-processing

Samples not meeting quality control standards defined by SomaLogic® (acceptance criteria concentration range 0.4–2.5 based on experimental controls) were removed from further analysis. For classifier discovery, 1129 protein assays were used, which included multiple isoforms for a single protein in some cases. For differential regulation and enrichment analysis, 3381 assays were used, with each protein represented once. The per-cohort sample and assay pass rate summary is included in Supplementary Table 1. Potential experimental confounders for these cohorts were checked for significant effects (e.g. assay plate, location, submission box, cohort and submission batch year). Principal component analysis was used to understand underlying variation and where confounders were related to principal components, a further step was introduced. Visual inspection was used to assess whether any of the recorded experimental variables were associated with any of the first principal components, highlighting cohort and array type as confounders. After this step, two different pre-processing strategies were used depending on the final objective. When the final objective was biomarker discovery, protein concentrations were normalized per data subset. When the final objective was differential expression analysis, protein concentrations were normalized using a bulk strategy.

### Targeted protein analysis

A narrative review of Parkinson’s disease biomarker studies was conducted to select a range of diagnostic and prognostic proteins. This resulted in a list of 50 proteins, although not all were represented in our SomaLogic® platform. Protein expression was log_2_ transformed. Finally, a list of 25 unique proteins (30 assays total) was taken forward for univariate analysis using a simple logistic regression (disease status) or linear regression (MoCA and MDS-UPDRS III) on each sample subset. As this method focused on selected proteins to test known biomarkers, *P*-values are presented as independent results without adjustment.

### Classification of a biomarker

#### Biomarker method quality control

The classification pipeline incorporated all the samples measured during the project, using only those proteins common to Versions 2 and 3 of the SomaLogic® platform (*n* = 1047). To allow the unbiased inclusion of all sample type modalities, we limited the selection to the 1047 proteins found on the Version 2 SomaScan® array. Sample sets were split by batch and cohort for initial quality checks before assignment into testing and training groups as described in Fig. 1. Each data set was corrected for unwanted variation due to demographics by building a generalized linear model per protein and then retaining the residuals calculated by the model. In this case, these generalized linear models used the to-be-corrected protein level as the outcome, while the predictor variables were the covariates. The covariates were cohort (Serum-1K only), participants’ LEDD at time of sampling, date samples were tested on the SOMAscan® assay (OPDC-CSF only) and the date the samples were prepared (Brain only). Following this correction, each data set was *Z*-score standardized.

#### Discovery and classification

We first performed a multi-stage ML discovery analysis—training and testing—to identify a multi-protein signature of disease using a case–control design. In the OPDC-serum data set (training), we ranked all proteins using simulated annealing as implemented in the R package ‘caret’ (version 6.0.77).^19^ This iterative algorithm starts with a random selection of proteins, then randomly perturbs the selection and keeps the new list of proteins only if it is better at discriminating cases from controls with a generalized linear model. Random perturbation is repeated iteratively until the algorithm converges on a final list of proteins (1000 iterations). Proteins are then ranked according to how often they are selected for their discriminative power. In the following step, using the resulting ‘N’ top-ranked proteins, a random forest classifier was built (R package ‘caret’) to discriminate between cases and controls. This was repeated with increasing values of N, from 1 to 200, creating a total of 200 random forest classifiers and recording in each case the accuracy that the classifier achieved.

For the testing stage, the identified protein classifiers were assessed in three independent data sets, each representing three different tissue types, serum (Serum-1K), CSF (OPDC-CSF) and post-mortem brain (Brain). The classifier output from the replication stage was taken as a refined protein panel set.

Classifiers were compared using the area under the receiver operator characteristic (ROC) curve, which was calculated using the function ‘predict’ as implemented in the R base package ‘stats’, the ‘prediction’ and ‘performance’ functions as implemented in the R package ‘ROCR’ (version 1.0.7). The *P*-value associated with this area under curve (AUC) was calculated using 10 000 random permutations of disease status to create a distribution of the statistic. Optimizing biomarker discovery methodology in a multi-modal cohort is a key to finding the set of proteins that most robustly predict the outcome. Our biomarker was required to perform well in all modalities and therefore, selection of the correct sample set for optimization was important. The three sample sets were used for a basic training analysis, OPDC-serum, TP-serum and CSF (OPDC). The strongest AUCs after training were for CSF and brain (Supplementary Table 2). However, the priority for this analysis was a good prediction AUC in a serum sample set. The serum training set selected was from a single cohort (OPDC) and contained similar case–control proportions to the testing data sets (70–30%).

### Replication of the novel multi-protein signature

The best multi-protein classifier, or signature, was then explored further in the replication data set, which consisted of serum samples from TP. Protein expression data was log_2_ transformed as a standard experimental normalization strategy. The two approaches were used to convert the signatures from the protein classifier to a single indicator. First, a mean expression per sample with a *Z*-score transformation was used. Secondly, singular value decomposition was used to generate an eigengene representation of the signature in an approach to reduce dimensionality. The performance of these classifiers or signatures in CSF (total protein = 142), brain (total protein = 89) and serum (*n* = 31) were assessed using association with disease status as an outcome.

### Protein differential regulation and enrichment analysis

In order to explore disease mechanisms, we focused on the data generated from the Version 3 SomaLogic® assay, which included 4006 protein assays. Protein expression values were log_2_ transformed. This analysis used 1599 serum samples with a matched case–control structure from the two longitudinal cohorts. An additional quality control step was undertaken to reduce between-cohort effects. ComBat normalization^20^ was employed to remove this structure, while measuring data set structure was checked using uniform manifold approximation and projection clustering. We performed differential regulation analysis on log_2_ transformed data using linear models corrected for age (continuous), sex and cohort. All generalized linear models were created using the R function ‘glm’ with a linkage function of binomial for disease status and Gaussian for continuous phenotypes (e.g. MoCA and MDS-UPDRS). For pathway enrichment analysis over-representation analysis (ORA) was implemented in the R library clusterProfiler^21^ using the KEGG (Kyoto Encyclopedia of Genes and Genomes) and Gene Ontology databases whereby proteins signatures are tested (hypergeometric) to understand whether a function or pathway is enriched more than would be expected by chance.

Comprehensive co-expression analysis was performed using weighted gene correlation network analysis (WGCNA).^22^ The standard pipeline was adapted for use with the proteins as described by Seyfried *et al*.^23^ Briefly, modules were capped at a minimum protein number = 30, power at 2 and the block-wise adjustment step was incorporated to reduce module noise and increase the robustness of protein selection. ORA was implemented on each module to understand functionality and relevance to disease mechanism. The eigengene value for each module was compared with clinical measures using logistic regression (Parkinson’s disease case status) and a general linear model (MoCA and MDS-UPDRS III) to understand the relationship to disease.

### Statistical analysis

All data pre-processing and analysis was performed using the statistical coding language R (version 4.0.2), with specific library choices described with each method. For targeted regression analysis, a *P* < 0.05 was applied to assess significance. For pathway analysis, a more conservative level was required and adjusted *P*-values were calculated according to Benjamini–Hochberg. A 95% confidence interval (CI) is reported for each analysis in addition to *P*-values.

## Results

Using an aptamer capture assay measuring over 1000 proteins, we sought to find signatures of disease that might function as biomarkers and provide insights into disease mechanisms. To do this, we utilized serum samples from two cohorts, the OPDC and TP, together with CSF samples from the OPDC and brain samples from the PUKBB. The participant characteristics from these data sets are described in Table 1. The ratio of cases to controls across samples varied from 82 to 65%, with cases predominating. As expected, there was a male predominance and the mean age for the PUKBB was from 9 to 13 years older.

**Table 1:** Demographics for a three cohort study of proteomic expression

	OPDC-Serum	OPDC/TP-1K	PUKBB-Brain	OPDC-CSF	TP-Serum
Total samples	572	224	37	85	932
Disease status Case (%) Control	400 (69.9) 172 (30.1)	159 (71) 65 (29)	24 (64.9) 13 (35.1)	70 (82.4) 15 (17.6)	671 (81.9) 148 (18.1)
Sex (%) Male Female	326 (57) 246 (43)	161 (71.9) 63 (28.1)	29 (78.4) 8 (21.6)	53 (62.4) 32 (37.6)	567 (60.8) 365 (39.2)
Cohort (%)	OPDC (100)	TP (35.7) OPDC (64.3)	PUKBB (100)	OPDC (100)	TP (100)
Mean age at sampling [SD] (range)	All 66.8 [10.4] (28.5–90.9) Cases 68.6 [9.6] (32.2–90.9) Controls 62.4 [10.9] (28.5–88.9)	All 70.4 [6.5] (53.6–83.5) Cases 70.4 [6.4] (53.6–83.5) Controls 70.6 [6.7] (54.7–83.4)	All 78.9 [8.2] (58–91) Cases 79.2 [6] (63–90) Controls 78.2 [11.4] (58–91)	All 65.9 [8.6] (39.7–85.1) Cases 65.8 [8.8] (39.7–83.6) Controls 66.1 [7.9] (55.3–85.1)	All 66.4 [9.3] (33.5–87.4) Cases 66.9 [8.8] (37–85.6) Controls 62.1 [9.7] (36.9–87.4)
Mean LEDD dosage at time of sampling* [SD] (range)	352.9 [263.9] (0–1450)	302.1 [197.5] (0–1310)	403.5 [324.2] (0–1100)	326.3 [258.3] (0–1120)	283.3 [200.6] (0–1380)
Mean MoCA score at sampling [SD] (range)	All 25 [3.5] (10–30) Cases 24.2 [3.5] (10–30) Controls 26.8 [2.7] (15–30)	All 24.3 [4.7] (10–30) Cases 23.8 [5.1] (10–30) Controls 25.7 [3.1] (16–30)	-	All 25.7 [3.1] (17–30) Cases 25.3 [3.1] (17–30) Controls 27.5 [2.1] (23–30)	All 25.1 [4.1] (0–30) Cases 25.3 [3.2] (13–30) Controls 24.4 [7.1] (0–30)
Mean UPDRS3 score at sampling [SD] (range)	All 20 [15.3] (0–78) Cases 27.8 [11.3] (4–78) Controls 1.8 [2.9] (0–19)	All 19.8 [18.2] (0–74) Cases 26.8 [16.9] (1–74) Controls 2.3 [2.9] (0–13)	-	All 22.9 [14.4] (0–74) Cases 27.4 [11.6] (10–74) Controls 2.3 [2.9] (0–9)	All 19.7 [13.3] (0–70) Cases 22.5 [12] (1–70) Controls 3.6 [5.4] (0–38)

MoCA and UPDRS III score not available for brain bank samples.

CSF, cerebrospinal fluid; LEDD, levodopa equivalent daily dose; OPDC, Oxford Parkinson’s Disease Centre; TP, tracking Parkinson's; PUKBB, Parkinson’s UK Brain Bank; SD, standard deviation. *LEDD values for cases only.

### Comparing biomarker predictions in different sample modalities

**Figure 2:**
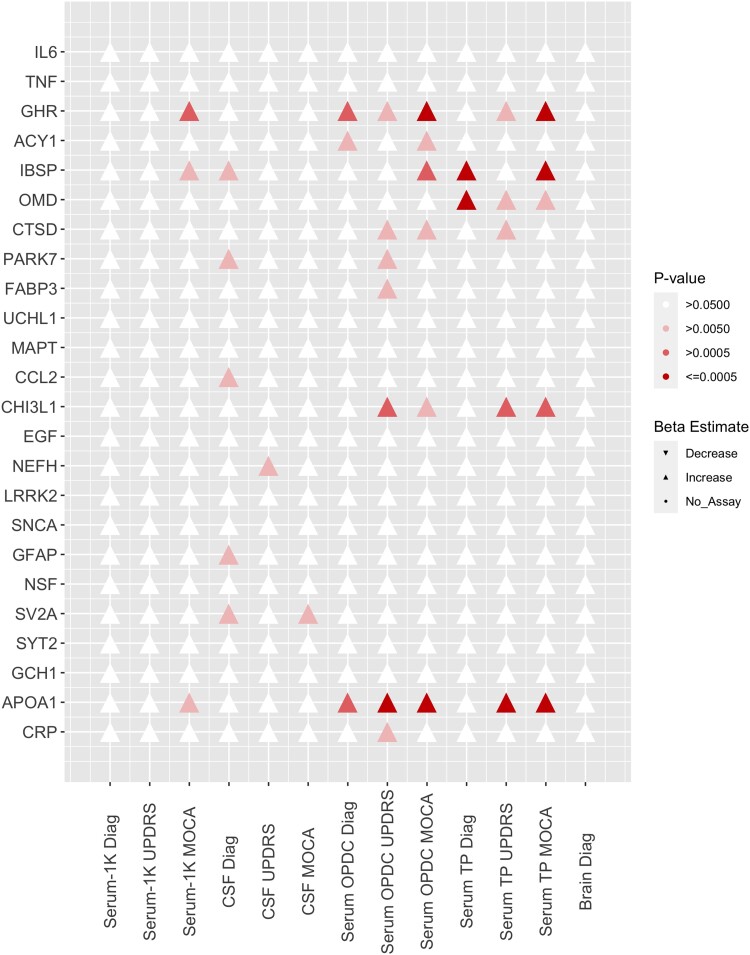
**Targeted biomarker protein results.** A significance matrix representing the association between protein expression of known biomarkers and Parkinson’s disease phenotypes across the project data sets. Univariant logistic regression was used for diagnosis (disease status). Univariant linear regression was used for MoCA (cognition) and MDS-UPDRS (movement) scores. The direction of effect is shown by up (positive) and down (negative) triangles.

We first wanted to assess whether putative biomarkers, previously suggested by a range of published studies, could be replicated using our proteomic data. From a list of 50 Parkinson’s disease-specific, neurodegenerative and cell-type markers, 25 were included in assays on the SomaLogic® panel. Apolipoprotein A1 and growth hormone receptor protein expression were related to at least one disease phenotype in all the serum sample sets tested (Fig. 2). Furthermore, bone sialoprotein (IBSP) was associated with the MoCA phenotype in all the serum data sets. In the CSF sample set, five suggested biomarker proteins showed an association with disease status (*P* > 0.05). These included glial fibrillary acidic protein (GFAP), IBSP and DJ-1. None of the proteins tested in brain tissue was related to the disease. Interestingly, no proteins tested were significantly related to the Parkinson’s disease phenotypes in every modality. These results suggest that there is scope for improvement of single protein biomarkers, perhaps instead as part of a multi-protein panel. Therefore, the next objective was to discover new multi-protein combinations that could be utilized as biomarkers.

### Defining a robust proteomic biomarker using multi-modal cohorts

**Figure 3:**
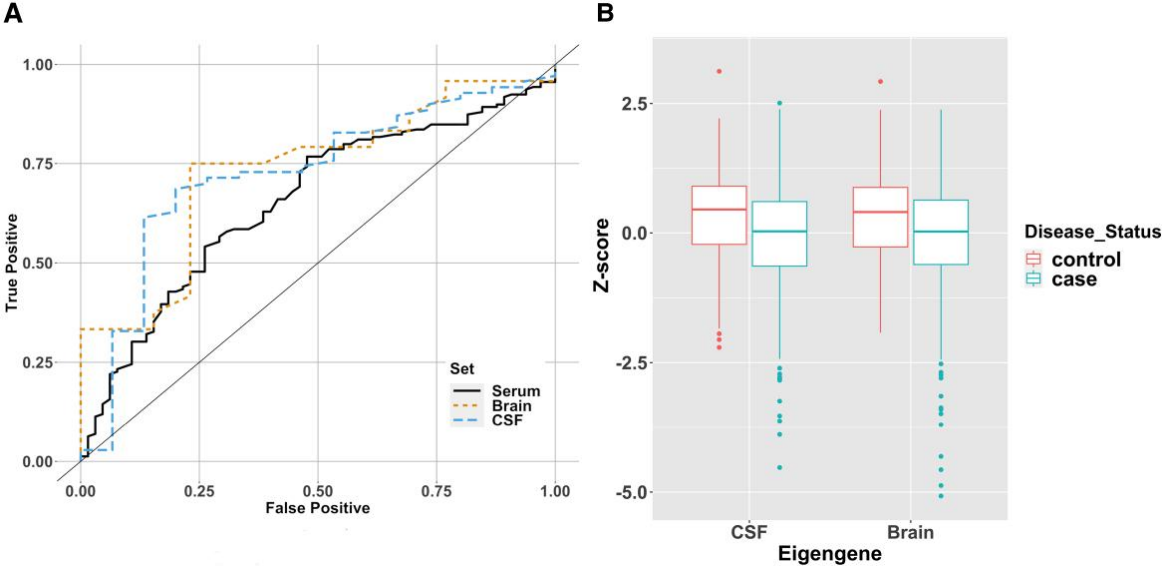
**Protein classifier testing showed strong potential biomarkers in both CSF and brain sample sets.** (**A**) ROC curve for each of the best classifiers when testing in the sample sets (CSF AUC = 0.74, *P* = 0.0009; brain AUC = 0.75, *P* = 0.006; serum AUC = 0.66, *P* = 0.0002). The black line defines what to expect by chance from a classifier. From left to right, an ideal classifier would follow a line up the *y*-axis and then along the *x*-axis, which would have an AUC of 1. (**B**) Derived classifier eigengenes are significantly associated with disease status in an independent serum data set (CSF: β = −0.44, *P* = 7.06 × 10^−6^; Brain: β = −0.42, *P* = 2.61 × 10^−5^; logistic regression).

Given the limited replication of previously identified biomarkers, which suggested that a known biomarker signal is detectable but perhaps not fully transferable between modalities, we went on to generate a *de novo* signature of disease from all 1004 proteins captured by the SomaLogic® data common to all three sample sources in this study. We applied ML approaches to first prioritize protein selection from a training set in serum (*n* = 572) and then carry out testing in serum (*n* = 227), CSF (*n* = 45) and brain (*n* = 37) samples. Briefly, we used simulated annealing and random forest for protein selection in the training serum set (OPDC—serum), which produced 200 potential classifiers, each using 1–200 of the top-ranked proteins. In the next stage, we tested each classifier in all three modalities to understand their performance in other tissue types: post-mortem brain (PUKBB—brain), CSF tissue (OPDC—CSF) and the serum (OPDC/TP-1K). Figure 3A shows a comparison between classifiers generated on different modality test sets. The brain sample proteins had an AUC of 0.75 (*P* = 0.006), CSF AUC was 0.74 (*P* = 0.0009) and serum AUC was 0.66 (*P* = 0.0002). These classifiers used the top 31, 86 and 142 ranked proteins, respectively (Table 2). Although similar in AUC values, the CSF had a reduced 95% CI compared with the brain classifier (Supplementary Fig. 1). Despite selecting from the same 200 proteins the serum sample set was optimal at 31 proteins. The best classifier outcomes, optimized in the CSF samples, included APOE and GFAP (Supplementary Table 3). Enrichment analysis of the CSF classifier proteins detected four KEGG pathways, including cytokine–cytokine receptor interaction (adjusted *P* = 0.004) as well as complement and coagulation cascades (adjusted *P* = 0.041). Using the smaller brain classifier protein list, only a single KEGG pathway was found; cytokine–cytokine receptor interaction (adjusted *P* = 0.0002).

**Table 2:** Filtering results of top-ranked proteins using three modalities

Data set	Number of proteins (classifiers)	AUC (95% CI)	*P*-value
***Testing classifier on three modalities to select best predictive proteins***			
Serum	31	0.66 (0.59–0.74)	2.00 × 10^−04^
Brain	86	0.75 (0.57–0.93)	0.0063
CSF	142	0.74 (0.61–0.87)	9.00 × 10^−04^
***Testing classifier on different disease outcomes to identify proteins predictive of disease severity***			
Severe	31	0.72 (0.63–0.81)	1.0 × 10^−4^
Intermediate	18	0.72 (0.61–0.83)	2.0 × 10^−4^
Mild	12	0.63 (0.53–0.72)	0.0063

### Replication of the novel protein panel in an independent cohort

Finally, we sought to replicate the best of the classifiers using a set of serum samples (*n* = 932) from the TP cohort that had not been included in the prior training and testing process. Given the similarity between the AUC (0.74 and 0.75) of the CSF and brain classifiers, we took both forward for further analysis. Initially, we used exploratory methods to understand how the classifier proteins related to each other. Focusing first on the larger protein signature, protein expression was tested between protein correlations to understand the range and representation. Supplementary Fig. 2 shows the clustering of the correlation values to both illustrate the similarities between subgroups of proteins in the signature and to demonstrate the range of protein concentration selected to differentiate between cases and controls. In order to replicate the complex signature of the disease, we converted the classifiers to a single indicator or eigengene. This single-value expression signature was calculated from a mean per sample as well as a dimensional reduction approach to produce an eigengene per sample. These single-value expression signatures were compared with clinical outcomes (Fig. 3B). Both the CSF and brain classifiers showed higher values and were significantly associated with diagnosis status using both signature methods (*P* < 0.001, Supplementary Tables 4 and 5), providing further confirmation.

### Understanding the effects of disease severity on biomarker discovery

To understand the effects of disease severity, we then compared the effects of the protein signature in a defined subgroup of patients with relatively severe and mild disease phenotypes. We tested our 200 protein classifier (as defined previously) in three serum sample subsets (severe, intermediate and mild). These independent serum classifiers demonstrated good predictive performance in finding the difference between the outcome samples set as compared with the control group (*P* < 0.01, Table 2). The severe outcome subgroup had an AUC of 0.72 with a selection of 31 proteins. This is an improvement compared with the AUC of 0.66 for the complete serum disease cohort. These 31 proteins were enriched only for the cytokine–cytokine receptor interaction; 12 of the proteins were in this KEGG pathway (adjusted *P* = 0.0018).

As the strongest classifier, the severe outcome signature was explored further in the independent serum replication set (TP). We compared both the severe signature (22 proteins passing cohort-level quality control) and the mild signature (nine proteins only) to the Parkinson’s disease phenotypes to understand whether these proteins reflected disease severity in an alternative data set. The MDS-UPDRS III and severity scores were significantly associated with both severe (beta = −1.31, *P* = 0.003 and beta = 0.14, *P* = 0.025) and mild phenotype protein signatures (beta = −1.49, *P* > 0.001 and beta = −0.20, *P* > 0.001). There were no significant associations between MoCA and either severity phenotype signature (Supplementary Table 6). Although the nine proteins in the mild signature did not show any pathway enrichment, they were more strongly related to the disease phenotypes tested.

### Differential expression of key proteins demonstrates a relationship to both known and novel Parkinson’s disease mechanisms

Having identified a possible biomarker signature from the 1004 proteins common across the samples we used, we then went on to explore protein differences using the full 4001 proteins in samples analysed with Version 4 of the SomaLogic® panel. A total of 1599 serum samples were analysed with the Version 4 array, of which 3378 proteins passed QC in all samples.

**Figure 4:**
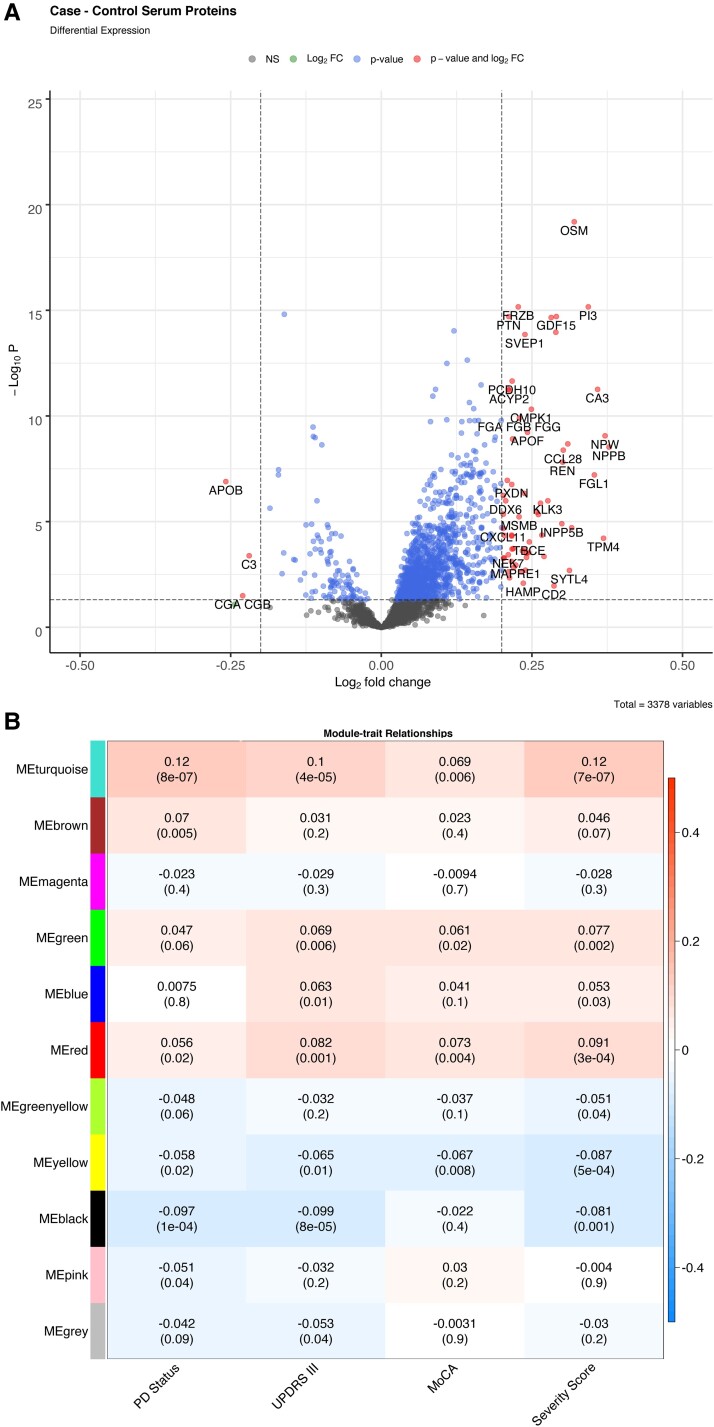
**Differential regulation and WGCNA summary from serum protein expression.** (**A**) Fold change plot showing genes with differential regulation between Parkinson’s disease cases versus control. (**B**) WGCNA module eigengenes correlation comparison with Parkinson’s disease traits.

We performed univariable regression to ascertain the significance of each protein individually. This was implemented for each of the outcome variables; disease status (case versus control), cognitive decline (MoCA score) and motor symptoms (MDS-UPDRS III score). The greatest changes in protein expression were found when comparing the disease status of the samples and this resulted in 261 differentially expressed proteins used for pathway analysis (Fig. 4A). In addition, 89 proteins were associated with a motor function (MDS-UPDRS III) and 13 with cognition (MoCA). Three proteins were associated with all phenotypes: oncostatin-M (OSM); complement component 9 (C9) and carnosine dipeptidase 1.

To understand whether the changes in protein could potentially show changes in function, we performed an enrichment analysis. Pathway analysis showed that the main significantly dysregulated protein pathways of case status were the complement and coagulation cascades (*P* = 0.009) and protein digestion and absorption (*P* = 0.015). The fat digestion and protein absorption pathway were enriched for dysregulated proteins associated with motor symptoms as measured using the MDS-UPDRS III (*P* = 0.001).

**Figure 5:**
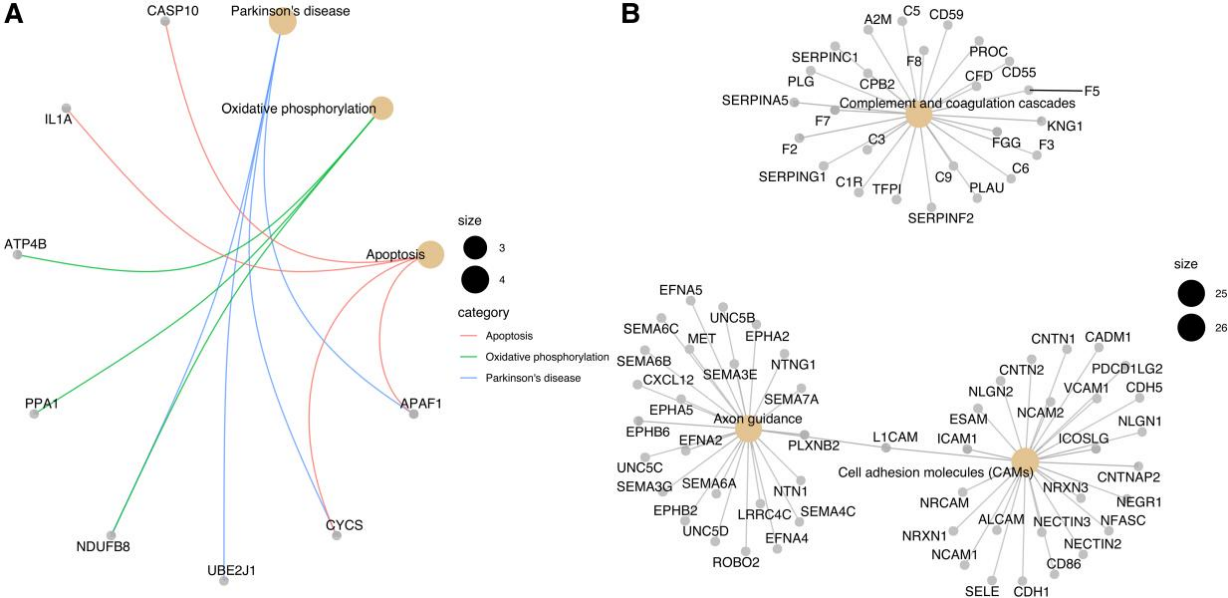
**Pathway enrichment from serum protein expression.** (**A)** MEyellow module protein network with known Parkinson’s disease protein pathway focus. (**B)** The MEturquoise protein network includes pathways with immune function and axon guidance.

We then used co-expression analysis with WGCNA to identify modules of differential protein expression associated with disease status within each case and a protein eigengene to represent that module expression. The four module eigengenes were significantly correlated to all Parkinson’s disease phenotypes (Fig. 4B, *P* < 0.05). The yellow module eigengene (MEyellow) was negatively correlated with the tested traits. It contained only 98 proteins and had a relationship directly to Parkinson’s disease, oxidative phosphorylation and apoptosis pathways (*P* < 0.05, Fig. 5A). The largest module (MEturquoise) was positively correlated to the traits and had 704 proteins and three key disease pathways, including complement, coagulation cascades and axon guidance (Fig. 5B). Interestingly, the red module was enriched for immune pathways in both the GO and KEGG databases (Supplementary Table 2). The green module had little apparent functional relevance. Table 3 shows details for the three significant disease-related modules. Each module has a hub protein, which has the strongest association with the rest of the module. The MEturquoise module is represented by the neurogenic locus notch homologue protein 1 precursor (NOTCH1) protein, part of the notch signalling pathway, which is involved in vascular development and angiogenesis. The 15-hydroxyprostaglandin dehydrogenase (HPDG), the hub protein for the red module is part of the prostaglandin signalling pathway.

**Table 3:** WGCNA protein modules with endophenotype relationships

	Parkinson’s disease Status (*P*-value)	UPDRS (*P*-value)	MOCA (*P*-value)	Severity score (*P*-value)	Total proteins	Hub proteins	Enriched KEGG pathways
MEred	0.034	0.001	0.004	0.0003	142	APEX1 DNA−(apurinic or apyrimidinic site) lyase (APEX1)	Spliceosome
MEturquoise	4.09 × 10^−7^	4.63 × 10^−5^	0.007	6.79 ×10^−7^	704	Neurogenic locus (NOTCH1)	Complement and coagulation cascades, axon guidance
MEyellow	0.012	0.011	0.008	0.0005	98	HPGD 15-HPDG [NAD(+)]	Parkinson’s disease, oxidative phosphorylation, apoptosis

Modules with significant correlation with all disease phenotype were selected for further analysis. The three modules described passed *P*-value thresholds (adjusted *P* < 0.05) and had functional relevance.

## Discussion

### Key results

We report here on a large biomarker discovery programme, including both unsupervised and confirmatory analyses using three different modalities (serum, CSF and brain tissue) and three different cohorts with a panel of aptamer capture protein assays, including over 1000 and 4000 proteins in different phases of the study.

The first analysis generated a novel signature of proteins associated with Parkinson’s disease. We used an ML approach to identify a protein signature differentiating case samples from control subjects. The resulting classifier was tested on two different cohorts to produce a more robust signature. Testing was also performed across tissue modalities to produce a more representative signature of disease (AUC = 0.66–0.75). Reproducibility was a priority for study design hence the inclusion of a comprehensive serum replication set where the protein signature had a significant association to Parkinson’s disease status and movement phenotypes (*P* < 0.001). This final signature contained proteins from the cytokine–cytokine receptor interaction pathway as well as the complement pathway. Differential regulation and clustering of protein expression provided further evidence for the enrichment for the complement pathway in Parkinson’s disease; a key element of the innate immune response that appears to be a common feature of multiple neurodegenerative diseases.^24^ Findings are consistent with previous work from the OPDC cohort showing that the most severely affected baseline Parkinson’s disease subtype was associated with a proinflammatory profile, comprising raised C-reactive protein and reduced APOEA1.^25^

The AUC values reported here are modest and lower than would be hoped for a simple blood-based biomarker to be used in clinical practice, albeit the CSF values were reasonable. However, the fact that we show robust replication and replication across multiple tissue types and cohort sources demonstrates that a blood-based signature of Parkinson’s disease is an achievable objective. Moreover, the fact that we identify pathways and processes known to be involved in neurodegeneration, including Parkinson’s disease, strongly supports the contention that this signature is biologically relevant. Further work will be needed to identify the optimal components of this signature for use in practice and clinical research and technological enhancements of future arrays may have better predictive value.

A biomarker would have the most clinical utility if it could be used for early, ideally preclinical, identification of disease or if it could be used as a surrogate for more specific markers of disease pathology. In the related field of Alzheimer’s disease biomarkers, the availability of definitive markers of disease pathology such as Positron emission tomography (PET) scans and CSF markers of both amyloid and tau pathology have enabled such studies, e.g. PET Amyloid can be used as an endpoint or outcome variable for biomarker discovery.^13,14^ However, such designs are not yet possible in Parkinson’s disease—there is no PET radioligand or CSF assay of alpha-synuclein to act as the equivalent anchor point. In the absence of such *in vivo* markers of disease pathology, we used a design where we compared different stages of disease using measures of motor and cognitive progression in the hope that a change in biomarker across people with symptomatic disease could be extrapolated to very early or prodromal disease and potentially to truly preclinical disease. Using this approach to describe the phenotypes and building on the ML methods, we generated a serum protein signature tested in severe, mild and intermediate patient subsets. The classifiers had an increased AUC (0.72) compared with the ungrouped serum samples (0.66), suggesting a stricter disease phenotype is better for disease prediction and therefore refining the proteins included in the signature.

The signature for ‘severe’ disease included particular proteins related to cytokine receptor enrichment, which constituted nearly half of the signature. These proteins were associated with both severity and motor phenotypes in the replication analysis. Although we do not have a clear biomarker for preclinical changes, we have been able to use this approach to understand more about the involvement of key proteins in disease progression. Further studies to examine this signature and its constituent proteins in early disease and in preclinical and prodromal Parkinson’s disease cases from longitudinal studies are warranted and will confirm if they can be used to predict the rate of conversion to a Parkinson’s disease clinical diagnosis.

There are several novel proteins described in these analyses, which are common to more than one approach. OSM was found in the CSF classifier and also detected in the analyses of differential regulation in relation to disease status, cognitive and movement scores. The known biomarkers APO1A^26^ and GFAP were present in the CSF classifier, which could act as positive controls for the signature. Using pathway enrichment approaches, we identified many Parkinson’s disease relevant pathways. The axon guidance pathway contains proteins related to synaptic dysfunction^27^ and the complement and coagulation cascades are an important pathway in innate immune responses previously identified in neurodegenerative diseases.^24,28^ Using co-expression clustering, we detected a module (MEturquoise) represented by the NOTCH1 protein, which has a reported Parkinson’s disease relationship as it is modulated by the LRRK2 protein.^29^ HPDG, the hub protein for the red module, is part of the prostaglandin signalling pathway with a role in neurodegeneration.

### Limitations

As in any observational cohort study, there are limitations to this work. We used two cohorts, one brain bank and three sample modalities, which, although they gave great power to detect associations, also introduced cohort and tissue noise. Without pathological confirmation, there is likely to be some misclassification due to cases of multiple system atrophy or progressive supranuclear palsy being wrongly included. This is likely to be a low rate as clinicians were all movement disorder specialists,^30^ red flags were used to exclude participants, we excluded those with a probability of Parkinson’s disease < 90% and follow-up allowed us to check for revised patient diagnoses. Additionally, we use here only neurologically healthy controls, making the task of disease diagnosis relatively simple compared with the clinically diverse data sets that we would hope a biomarker could perform in. However, despite the spectrum bias, we were still able to distinguish disease severity, which will aid in the end goal of prognostic signatures.

Using a data set, which has been built up over years has led to some data loss from batch control but particular restriction came from the use of the older, lower protein number SomaScan® array for some samples. However, the strength of validating a signature on a different array should not be overlooked and this gives a more robust final result.

### Interpretation

Here, we report a proteomic signature of Parkinson’s disease in the blood that replicates across cohorts and validates across tissue types. Although of modest predictive power, the significance and replicability of the result is encouraging in the search for biomarkers that could be used to enhance clinical research, specifically trials, to identify and recruit well-characterized participants to clinical trials. Deep proteomic analyses of over 4000 proteins identified disease pathways, including those of neuroimmunity and other known neurodegeneration-associated functions, suggesting that targeting some of these biological processes might be of value in disease modification therapeutics. Indeed, these results raise the possibility of selecting subgroups of people for clinical trials using a precision medicine approach. Interestingly, the overlap in disease process between Parkinson’s disease–associated proteins in this study and Alzheimer’s disease–associated proteins in similar studies in that disease again points to commonalities and overlaps between neurodegeneration suggesting that a precision medicine approach might not be possible but might be a necessary route to effective treatments for these devastating disorders.

## Conclusion

Using the largest and most comprehensive proteomics Parkinson’s disease data sets, we have described a range of approaches for biomarker discovery and confirmed the potential of large-scale data analysis. We have demonstrated the potential value of a multi-protein signature of novel markers for further analysis and replication in new large data sets as these become available.

## Supplementary Material

fcac343_Supplementary_Data

## Data Availability

Data are available on application to cohort owners.

## Abbreviations

AUC =: area under curve; KEGG = Kyoto Encyclopedia of Genes and Genomes
LEDD =: levodopa equivalent daily dose
MDS-UPDRS =: Movement Disorder Society-unified Parkinson’s disease rating scale
ML =: machine learning
MoCA =: montreal cognitive assessment
OPDC =: Oxford Parkinson’s Disease Centre
ORA =: over-representation analysis
PRoBaND =: Parkinson’s repository of biosamples and networked data sets
PUKBB =: Parkinson’s United Kingdom Brain Bank
TP =: Tracking Parkinson’s
WGCNA =: weighted gene correlation network analysis
