# Supplementary material for: Identification of a possible proteomic biomarker in Parkinson’s disease: discovery and replication in blood, brain and cerebrospinal fluid
Source: Brain Commun. 2022 Dec 28;5(1):fcac343. doi: 10.1093/braincomms/fcac343 (PMC9856276; doi:10.1093/braincomms/fcac343)
Supplement: fcac343_Supplementary_Data [file fcac343_supplementary_data.docx]

# Supplementary Material - Identification of a possible proteomic Biomarker in Parkinson’s Disease: Discovery and Replication in Blood, brain and CSF

## Supplementary Methods - Classifying Disease Severity in the Cohorts

For the final phase of the analysis an equivalent approach was developed to generate a severity score per patient. The MoCA and MDS UPDRS III scores were standardised by square root transformation (Equation 1 A and B), then combined to generate a severity score per patient. This score was used to sort samples by being above or below the median.

$$\frac{\left( \sqrt{UPDRS3} \right)-4.5585}{1.2283}$$

$$\frac{\left( \sqrt{31-MOCA} \right)-2.2785}{0.6679}$$

## Supplementary Figure 1 - Receiver operator characteristic (ROC) curve comparing classifiers between testing sets

Receiver operator characteristic (ROC) curve for each of the best classifiers when testing in the ‘Brain’, ‘CSF’ and ‘Serum’ sample sets (CSF AUC = 0.74, p-value = 0.0009; brain AUC = 0.75, p-value = 0.006; serum AUC = 0.66, p-value = 0.0002). Shaded regions are represent 95% CI for each curve. Although similar in highest AUC value, the CSF sample set has a smaller AUC CI region.


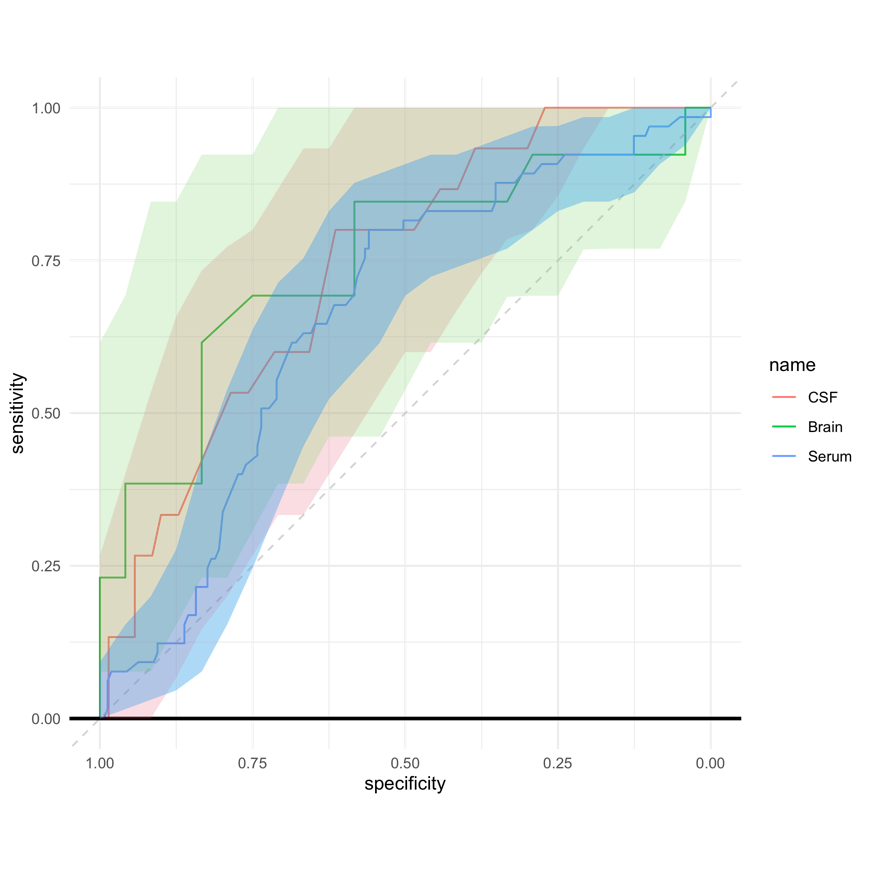


**Supplementary Figure 2 - Heatmap of protein classifiers expression in both CSF and brain sample sets**

Exploration of the signature protein expression was performed in independent TP - Serum set. Spearman’s correlation between expression of the CSF classifier proteins is shown by heatmap where proteins with similar concentration profiles are clustered together. Rho value correlations plotted where p-values < 0.05. Proteins present in both brain and CSF signatures are coloured yellow. Proteins unique to the larger CSF classifier are tagged with blue.


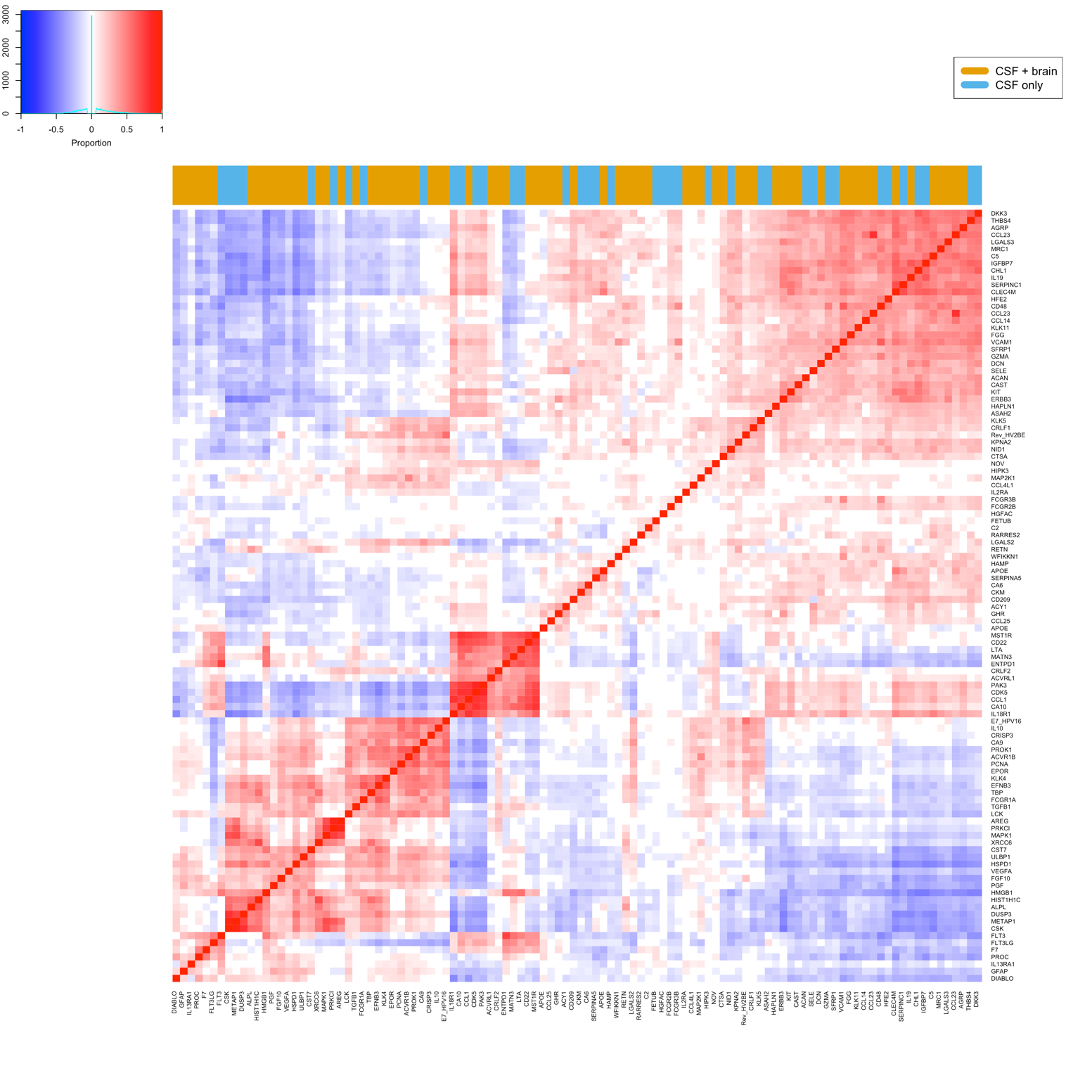


## Supplementary Table 1 - Per Cohort Samples passing Experimental Quality Control Checks

Batch based quality control was implemented before incorporating samples into analysis datasets.

| **Data Batch** | **Total samples assayed** | **Total samples passing QC** | **Total probes** | **Total probes passing Somalogic QC** |
| --- | --- | --- | --- | --- |
| OPDC/TP Serum 1K | 267 | 265 | 1129 | 1129 |
| OPDC - CSF | 40 + 45 | 40 + 45 | 1129 | 1129 |
| PDUKBB - Brain | 37 | 37 | 1129 | 1129 |
| OPDC - Serum | 623 | 619 | 4006 | 3964 |
| TP - Serum | 1030 | 932 | 4006 | 3381 |

TP = Tracking Parkinson’s, OPDC = Oxford Parkinson’s Disease Cohort, PUKBB = Parkinson’s Disease UK Brain Bank

## Supplementary Table 2 - Comparing Modalities for Optimising Data Training

We defined our classifier in serum and then used all modalities in our replication and refinement step. This was based objective assessment of the analysis plan. However, we performed a comparison to support this selection. Three sample sets were tested, OPDC serum, TP serum and CSF (OPDC). The strongest AUC after training were for CSF and brain. However, the priority for this analysis was for a good prediction AUC in serum testing. The serum training set selected was from a single cohort (OPDC) and contained similar case-control proportions to the testing datasets (70 - 30 %). By using the other modalities to reduce noise we have selected proteins which work in blood - one of our key objectives.

|  |  | **Testing Sets** | | | | | | | | | |
| --- | --- | --- | --- | --- | --- | --- | --- | --- | --- | --- | --- |
|  |  | **Serum - OPDC** | | **Serum - TP** | | **CSF - OPDC** | | **Brain** | | **Serum - 1K** | |
|  |  | **AUC** | **Classifier Total** | **AUC** | **Classifier Total** | **AUC** | **Classifier Total** | **AUC** | **Classifier Total** | **AUC** | **Classifier Total** |
| **Training Sets** | **Serum - OPDC** | X | X | 0.6 | 158 | 0.74 | 142 | 0.75 | 86 | 0.66 | 31 |
|  | **Serum - TP** | 0.51 | 3 | X | X | 0.75 | 86 | 0.64 | 48 | 0.54 | 1 |
|  | **CSF - OPDC** | 0.54 | 12 | 0.53 | 3 | X | X | 0.63 | 54 | 0.54 | 3 |

TP = Tracking Parkinson’s, OPDC = Oxford Parkinson’s Disease Cohort

## Supplementary Table 3 - Classifier Signature Proteins

| **Somalogic Protein Aptamer Target** | **Target Full Name** | **UniProt** | **Entrez Gene Symbol** | **Somalogic SeqID Identifier** | **Brain** | **Serum** | **Severe** | **Mild** |
| --- | --- | --- | --- | --- | --- | --- | --- | --- |
| a2-HS-Glycoprotein | Alpha-2-HS-glycoprotein | P02765 | AHSG | 3581-53_3 |  |  |  |  |
| ABL1 | Tyrosine-protein kinase ABL1 | P00519 | ABL1 | 3341-33_4 |  |  |  |  |
| Activin RIB | Activin receptor type-1B | P36896 | ACVR1B | 2806-49_2 | X |  |  |  |
| Aggrecan | Aggrecan core protein | P16112 | ACAN | 3280-49_2 |  |  |  |  |
| Albumin | Serum albumin | P02768 | ALB | 3707-12_2 |  |  |  |  |
| ALK-1 | Serine/threonine-protein kinase receptor R3 | P37023 | ACVRL1 | 2969-11_1 | X |  |  |  |
| Alkaline phosphatase, bone | Alkaline phosphatase, tissue-nonspecific isozyme | P05186 | ALPL | 2795-23_3 | X |  |  |  |
| ALT | Alanine aminotransferase 1 | P24298 | GPT | 3709-4_2 |  |  |  |  |
| Aminoacylase-1 | Aminoacylase-1 | Q03154 | ACY1 | 3343-1_4 |  |  |  |  |
| ANGL3 | Angiopoietin-related protein 3 | Q9Y5C1 | ANGPTL3 | 3281-19_1 |  |  |  |  |
| Antithrombin III | Antithrombin-III | P01008 | SERPINC1 | 3344-60_4 |  |  |  |  |
| Apo A-I | Apolipoprotein A-I | P02647 | APOA1 | 2750-3_2 | X | X | X |  |
| Apo E | Apolipoprotein E | P02649 | APOE | 2418-55_9 | X | X | X | X |
| Apo E4 | Apolipoprotein E (isoform E4) | P02649 | APOE | 2938-55_2 | X |  |  |  |
| AREG | Amphiregulin | P15514 | AREG | 2970-60_2 | X |  |  |  |
| ART | Agouti-related protein | O00253 | AGRP | 2813-11_2 | X |  |  |  |
| ASAH2 | Neutral ceramidase | Q9NR71 | ASAH2 | 3212-30_3 |  |  |  |  |
| Azurocidin | Azurocidin | P20160 | AZU1 | 2751-16_2 | X | X | X |  |
| BMP-14 | Growth/differentiation factor 5 | P43026 | GDF5 | 2752-62_2 | X | X | X |  |
| Bone proteoglycan II | Decorin | P07585 | DCN | 2666-53_2 | X | X | X |  |
| C1s | Complement C1s subcomponent | P09871 | C1S | 3590-8_3 |  |  |  |  |
| C2 | Complement C2 | P06681 | C2 | 3186-2_2 | X |  |  |  |
| C5a | C5a anaphylatoxin | P01031 | C5 | 2851-63_3 | X |  |  |  |
| Calpastatin | Calpastatin | P20810 | CAST | 3026-5_2 | X |  |  |  |
| Carbonic anhydrase 6 | Carbonic anhydrase 6 | P23280 | CA6 | 3352-80_3 |  |  |  |  |
| Carbonic anhydrase 9 | Carbonic anhydrase 9 | Q16790 | CA9 | 3798-71_1 |  |  |  |  |
| Carbonic Anhydrase X | Carbonic anhydrase-related protein 10 | Q9NS85 | CA10 | 3289-19_2 |  |  |  |  |
| Cathepsin A | Lysosomal protective protein | P10619 | CTSA | 3179-51_2 | X |  |  |  |
| CD22 | B-cell receptor CD22 | P20273 | CD22 | 2891-1_3 | X |  |  |  |
| CD39 | Ectonucleoside triphosphate diphosphohydrolase 1 | P49961 | ENTPD1 | 3182-38_2 | X |  |  |  |
| CD48 | CD48 antigen | P09326 | CD48 | 3292-75_1 |  |  |  |  |
| CDK1/cyclin B | Cyclin-dependent kinase 1:G2/mitotic-specific cyclin-B1 complex | P06493 P14635 | CDC2 CCNB1 | 3422-4_2 |  |  |  |  |
| CDK5/p35 | Cyclin-dependent kinase 5:Cyclin-dependent kinase 5 activator 1 complex | Q00535 Q15078 | CDK5 CDK5R1 | 3358-51_2 |  |  |  |  |
| CHL1 | Neural cell adhesion molecule L1-like protein | O00533 | CHL1 | 3601-54_3 |  |  |  |  |
| Ck-b-8-1 | Ck-beta-8-1 | P55773 | CCL23 | 3028-36_2 | X |  |  |  |
| CK-MB | Creatine kinase M-type:Creatine kinase B-type heterodimer | P12277 P06732 | CKB CKM | 3714-49_2 |  |  |  |  |
| CLF-1/CLC Complex | Cytokine receptor-like factor 1:Cardiotrophin-like cytokine factor 1 Complex | O75462 Q9UBD9 | CRLF1 CLCF1 | 2607-54_2 | X | X | X | X |
| Coagulation Factor VII | Coagulation factor VII | P08709 | F7 | 3184-25_2 | X |  |  |  |
| CRIS3 | Cysteine-rich secretory protein 3 | P54108 | CRISP3 | 3187-52_2 | X |  |  |  |
| CSK | Tyrosine-protein kinase CSK | P41240 | CSK | 3363-31_4 |  |  |  |  |
| CYTF | Cystatin-F | O76096 | CST7 | 3302-58_1 |  |  |  |  |
| DC-SIGN | CD209 antigen | Q9NNX6 | CD209 | 3029-52_2 | X |  |  |  |
| DC-SIGNR | C-type lectin domain family 4 member M | Q9H2X3 | CLEC4M | 3030-3_2 | X |  |  |  |
| DKK3 | Dickkopf-related protein 3 | Q9UBP4 | DKK3 | 3607-71_1 |  |  |  |  |
| DUS3 | Dual specificity protein phosphatase 3 | P51452 | DUSP3 | 3480-7_1 |  |  |  |  |
| EG-VEGF | Prokineticin-1 | P58294 | PROK1 | 2247-20_11 | X | X | X | X |
| Endothelin-converting enzyme 1 | Endothelin-converting enzyme 1 | P42892 | ECE1 | 3611-70_4 |  |  |  |  |
| Ephrin-B3 | Ephrin-B3 | Q15768 | EFNB3 | 2514-65_3 | X | X | X | X |
| EPO-R | Erythropoietin receptor | P19235 | EPOR | 2715-25_2 | X | X | X |  |
| ERBB3 | Receptor tyrosine-protein kinase erbB-3 | P21860 | ERBB3 | 2617-56_35 | X | X | X |  |
| FCG2B | Low affinity immunoglobulin gamma Fc region receptor II-b | P31994 | FCGR2B | 3310-62_1 |  |  |  |  |
| FCG3B | Low affinity immunoglobulin gamma Fc region receptor III-B | O75015 | FCGR3B | 3311-27_1 |  |  |  |  |
| FCGR1 | High affinity immunoglobulin gamma Fc receptor I | P12314 | FCGR1A | 3312-64_1 |  |  |  |  |
| FETUB | Fetuin-B | Q9UGM5 | FETUB | 3367-8_3 |  |  |  |  |
| FGF-10 | Fibroblast growth factor 10 | O15520 | FGF10 | 2441-2_4 | X | X | X | X |
| Fibrinogen | Fibrinogen | P02671 P02675 P02679 | FGA FGB FGG | 2796-62_2 | X |  |  |  |
| Flt-3 | Receptor-type tyrosine-protein kinase FLT3 | P36888 | FLT3 | 3437-80_3 |  |  |  |  |
| Flt3 ligand | Fms-related tyrosine kinase 3 ligand | P49771 | FLT3LG | 3053-49_2 | X |  |  |  |
| Galectin-2 | Galectin-2 | P05162 | LGALS2 | 3033-57_1 | X |  |  |  |
| Galectin-3 | Galectin-3 | P17931 | LGALS3 | 3066-12_1 | X |  |  |  |
| GFAP | Glial fibrillary acidic protein | P14136 | GFAP | 3034-1_2 | X |  |  |  |
| Glucocorticoid receptor | Glucocorticoid receptor | P04150 | NR3C1 | 2857-70_2 | X |  |  |  |
| granzyme A | Granzyme A | P12544 | GZMA | 3440-7_2 |  |  |  |  |
| Growth hormone receptor | Growth hormone receptor | P10912 | GHR | 2948-58_2 | X |  |  |  |
| HCC-1 | C-C motif chemokine 14 | Q16627 | CCL14 | 2900-53_3 | X |  |  |  |
| HGFA | Hepatocyte growth factor activator | Q04756 | HGFAC | 3617-80_4 |  |  |  |  |
| HIPK3 | Homeodomain-interacting protein kinase 3 | Q9H422 | HIPK3 | 3443-61_2 |  |  |  |  |
| Histone H1.2 | Histone H1.2 | P16403 | HIST1H1C | 2987-37_3 | X |  |  |  |
| HIV-2 Rev | Protein Rev_HV2BE | P18093 | Human-virus | 2769-3_2 | X | X | X |  |
| HMG-1 | High mobility group protein B1 | P09429 | HMGB1 | 2524-56_3 | X | X | X | X |
| HPLN1 | Hyaluronan and proteoglycan link protein 1 | P10915 | HAPLN1 | 3196-6_2 | X |  |  |  |
| HPV E7 Type 16 | Protein E7_HPV16 | P03129 | Human-virus | 2623-54_4 | X | X | X |  |
| HSP 60 | 60 kDa heat shock protein, mitochondrial | P10809 | HSPD1 | 2682-68_1 | X | X | X |  |
| I-309 | C-C motif chemokine 1 | P22362 | CCL1 | 2770-51_2 | X | X | X |  |
| IGFBP-7 | Insulin-like growth factor-binding protein 7 | Q16270 | IGFBP7 | 3320-49_2 |  |  |  |  |
| IL-10 | Interleukin-10 | P22301 | IL10 | 2773-50_2 | X |  |  |  |
| IL-13 Ra1 | Interleukin-13 receptor subunit alpha-1 | P78552 | IL13RA1 | 2633-52_2 | X | X | X |  |
| IL-16 | Interleukin-16 | Q14005 | IL16 | 2774-10_3 | X |  |  |  |
| IL-17B | Interleukin-17B | Q9UHF5 | IL17B | 3499-77_2 |  |  |  |  |
| IL-18 Ra | Interleukin-18 receptor 1 | Q13478 | IL18R1 | 3446-7_2 |  |  |  |  |
| IL-19 | Interleukin-19 | Q9UHD0 | IL19 | 3035-80_2 | X |  |  |  |
| IL-2 sRa | Interleukin-2 receptor subunit alpha | P01589 | IL2RA | 3151-6_1 | X |  |  |  |
| Kallikrein 11 | Kallikrein-11 | Q9UBX7 | KLK11 | 2831-29_1 | X |  |  |  |
| Kallikrein 4 | Kallikrein-4 | Q9Y5K2 | KLK4 | 2833-20_1 | X |  |  |  |
| kallikrein 5 | Kallikrein-5 | Q9Y337 | KLK5 | 3201-49_2 |  |  |  |  |
| Kallikrein 6 | Kallikrein-6 | Q92876 | KLK6 | 3450-4_2 |  |  |  |  |
| Karyopherin-a2 | Importin subunit alpha-1 | P52292 | KPNA2 | 2860-19_2 | X |  |  |  |
| KPCI | Protein kinase C iota type | P41743 | PRKCI | 3379-29_1 |  |  |  |  |
| Ku70 | X-ray repair cross-complementing protein 6 | P12956 | XRCC6 | 2835-1_4 | X |  |  |  |
| LAG-1 | C-C motif chemokine 4-like | Q8NHW4 | CCL4L1 | 2781-63_2 | X |  |  |  |
| LCK | Tyrosine-protein kinase Lck | P06239 | LCK | 3452-17_2 |  |  |  |  |
| LD78-beta | C-C motif chemokine 3-like 1 | P16619 | CCL3L1 | 2783-18_2 | X |  |  |  |
| LEAP-1 | Hepcidin | P81172 | HAMP | 3504-58_2 |  |  |  |  |
| Lymphotoxin a2/b1 | Lymphotoxin alpha2:beta1 | P01374 Q06643 | LTA LTB | 3506-49_1 |  |  |  |  |
| Macrophage mannose receptor | Macrophage mannose receptor 1 | P22897 | MRC1 | 2637-77_2 | X | X | X |  |
| MATN3 | Matrilin-3 | O15232 | MATN3 | 3208-2_4 |  |  |  |  |
| MEK1 | Dual specificity mitogen-activated protein kinase kinase 1 | Q02750 | MAP2K1 | 2864-2_3 | X |  |  |  |
| METAP1 | Methionine aminopeptidase 1 | P53582 | METAP1 | 3210-1_2 |  |  |  |  |
| MIP-3a | C-C motif chemokine 20 | P78556 | CCL20 | 2468-62_3 | X | X | X | X |
| MK01 | Mitogen-activated protein kinase 1 | P28482 | MAPK1 | 3115-64_2 | X |  |  |  |
| MMEL2 | Membrane metallo-endopeptidase-like 1 | Q495T6 | MMEL1 | 3627-71_4 |  |  |  |  |
| MMP-3 | Stromelysin-1 | P08254 | MMP3 | 2788-55_1 | X |  |  |  |
| MPIF-1 | C-C motif chemokine 23 | P55773 | CCL23 | 2913-1_2 | X |  |  |  |
| MRCKB | Serine/threonine-protein kinase MRCK beta | Q9Y5S2 | CDC42BPB | 3629-60_4 |  |  |  |  |
| MSP R | Macrophage-stimulating protein receptor | Q04912 | MST1R | 2640-3_2 | X | X | X |  |
| Myoglobin | Myoglobin | P02144 | MB | 3042-7_2 | X |  |  |  |
| NET4 | Netrin-4 | Q9HB63 | NTN4 | 3327-27_1 |  |  |  |  |
| Nidogen | Nidogen-1 | P14543 | NID1 | 3213-65_2 |  |  |  |  |
| NKp44 | Natural cytotoxicity triggering receptor 2 | O95944 | NCR2 | 2734-49_4 | X | X | X |  |
| NovH | Protein NOV homolog | P48745 | NOV | 2737-22_2 | X | X | X |  |
| NPS-PLA2 | Phospholipase A2, membrane associated | P14555 | PLA2G2A | 2692-74_2 | X | X | X |  |
| OSM | Oncostatin-M | P13725 | OSM | 2693-20_3 | X | X | X |  |
| PAK3 | Serine/threonine-protein kinase PAK 3 | O75914 | PAK3 | 3387-1_2 |  |  |  |  |
| PCI | Plasma serine protease inhibitor | P05154 | SERPINA5 | 3389-7_2 |  |  |  |  |
| PCNA | Proliferating cell nuclear antigen | P12004 | PCNA | 2915-6_2 | X |  |  |  |
| PlGF | Placenta growth factor | P49763 | PGF | 3078-1_2 | X |  |  |  |
| PRL | Prolactin | P01236 | PRL | 2585-2_5 | X | X | X | X |
| Protein C | Vitamin K-dependent protein C | P04070 | PROC | 2961-1_2 | X |  |  |  |
| PTP-1B | Tyrosine-protein phosphatase non-receptor type 1 | P18031 | PTPN1 | 3005-5_2 | X |  |  |  |
| RAP | alpha-2-macroglobulin receptor-associated protein | P30533 | LRPAP1 | 3640-14_3 |  |  |  |  |
| resistin | Resistin | Q9HD89 | RETN | 3046-31_1 | X |  |  |  |
| RGM-C | Hemojuvelin | Q6ZVN8 | HFE2 | 3332-57_1 |  |  |  |  |
| SARP-2 | Secreted frizzled-related protein 1 | Q8N474 | SFRP1 | 3221-54_1 |  |  |  |  |
| SCF sR | Mast/stem cell growth factor receptor Kit | P10721 | KIT | 2475-1_3 | X | X | X | X |
| SDF-1 | Stromal cell-derived factor 1 | P48061 | CXCL12 | 3516-60_2 |  |  |  |  |
| sE-Selectin | E-selectin | P16581 | SELE | 3470-1_2 |  |  |  |  |
| SMAC | Diablo homolog, mitochondrial | Q9NR28 | DIABLO | 3122-6_2 | X |  |  |  |
| TBK1 | Serine/threonine-protein kinase TBK1 | Q9UHD2 | TBK1 | 3400-49_2 |  |  |  |  |
| TBP | TATA-box-binding protein | P20226 | TBP | 2875-15_2 | X |  |  |  |
| TCPTP | Tyrosine-protein phosphatase non-receptor type 2 | P17706 | PTPN2 | 3401-8_2 |  |  |  |  |
| TECK | C-C motif chemokine 25 | O15444 | CCL25 | 2705-5_2 | X | X | X |  |
| TGF-b1 | Transforming growth factor beta-1 | P01137 | TGFB1 | 2333-72_1 | X | X | X | X |
| TIG2 | Retinoic acid receptor responder protein 2 | Q99969 | RARRES2 | 3079-62_2 | X |  |  |  |
| TNF sR-II | Tumor necrosis factor receptor superfamily member 1B | P20333 | TNFRSF1B | 3152-57_1 | X |  |  |  |
| tPA | Tissue-type plasminogen activator | P00750 | PLAT | 2212-69_1 | X | X | X | X |
| TSLP R | Cytokine receptor-like factor 2 | Q9HC73 | CRLF2 | 2746-56_1 | X | X | X |  |
| TSP4 | Thrombospondin-4 | P35443 | THBS4 | 3340-53_1 |  |  |  |  |
| ULBP-1 | NKG2D ligand 1 | Q9BZM6 | ULBP1 | 3081-70_2 | X |  |  |  |
| VCAM-1 | Vascular cell adhesion protein 1 | P19320 | VCAM1 | 2967-8_1 | X |  |  |  |
| VEGF | Vascular endothelial growth factor A | P15692 | VEGFA | 2597-8_3 | X | X | X | X |
| vWF | von Willebrand factor | P04275 | VWF | 3050-7_2 | X |  |  |  |
| WFKN1 | WAP, kazal, immunoglobulin, kunitz and NTR domain-containing protein 1 | Q96NZ8 | WFIKKN1 | 3191-50_2 | X |  |  |  |

## Supplementary Table 4 - CSF and Brain Classifier Signatures are associated with PD status in the validation dataset

| **Signature** | **Phenotype** | **Mean Estimate** | | | **Eigengene** | | |
| --- | --- | --- | --- | --- | --- | --- | --- |
|  |  | **Mean beta [CI]** | **Mean p** | **Mean AUC [CI]** | **SDV beta [CI]** | **SDV p** | **SDV AUC [CI]** |
| **CSF** | **Disease status** | 0.38  [0.19 - 0.57] | 1.05x10^-04^ | 0.61  [0.56 - 0.65] | -0.44  [-0.64 - -0.25] | 7.06 x10^-06^ | 0.62  [0.57 - 0.67] |
| **Brain** | **Disease status** | 0.33  [0.14 - 0.53] | 9.35 x10^-04^ | 0.59  [0.54 - 0.63] | -0.42  [-0.62 - -0.23] | 2.61 x10^-05^ | 0.61  [0.56 - 0.66] |

## Supplementary Table 5 - Comparison of regression methods to assess CSF and Brain Classifier Signatures

Logistic regression methods were tested to better understand signature performance using different approaches. Model 2 includes adjustment for age and gender. Model 3 includes a weighted propensity score. The propensity score was calculated using MatchIt (R library) used the link (probit) model, whereby age, sex and collection site were used to produce a weight per sample. Model 4 is presented as a comparison to discern the predictive power of age and sex independent from the protein signature (AUC = 0.67). Each signature was generated using two approaches, a mean estimate of protein expression (mean) and an eigengene (SDV).

|  |  | Beta [CI] | P - value | AUC [CI] |
| --- | --- | --- | --- | --- |
| Model 2 - Regression with adjustments | CSF Mean | 0.24 [0.03-0.46] | 0.0244 | 0.69 [0.45-0.94] |
|  | CSF SDV | -0.3 [-0.51--0.09] | 0.0064 | 0.68 [0.63-0.73] |
|  | Brain Mean | 0.24 [0.02-0.46] | 0.0356 | 0.69 [0.63-0.74] |
|  | Brain SDV | -0.31 [-0.53--0.09] | 0.0058 | 0.68 [0.63-0.73] |
| Model 3 - Regression with propensity score | CSF Mean | 0.36 [0.15-0.57] | 0.0004 | 0.6 [0.55-0.65] |
|  | CSF SDV | -0.4 [-0.61--0.19] | 6.91 x10^-05^ | 0.61 [0.56-0.66] |
|  | Brain Mean | 0.72 [0.49-0.97] | 6.37 x10^-08^ | 0.57 [0.52-0.63] |
|  | Brain SDV | -0.78 [-1.03--0.55] | 1.35 x10^-09^ | 0.6 [0.54-0.65] |
| Model 4: Regression with age and sex only | | 0.07 [0.05-0.1] | 4.04 x10^-10^ | 0.67 [0.62-0.72] |

## Supplementary Table 6 - Outcome Severity Classifier Signatures are associated with PD phenotypes

| **Signature** | **Phenotype** | **Mean estimate** | | | **Eigengene** | | |
| --- | --- | --- | --- | --- | --- | --- | --- |
|  |  | **Mean beta** | **Mean p** | **Mean CI** | **SDV beta** | **SDV p** | **SDV CI** |
| **Severe** | **Severity score** | -0.14 | 0.025 | -0.25 - -0.02 | 0.14 | 0.025 | 0.02 - 0.25 |
|  | **MOCA** | 0.01 | 0.916 | -0.2 - 0.22 | -0.02 | 0.846 | -0.23 - 0.19 |
|  | **UPDRS III** | -1.31 | 0.003 | -2.17 - -0.45 | 1.31 | 0.003 | 0.44 - 2.17 |
| **Mild** | **Severity score** | -0.20 | 7.58 x10^-04^ | -0.32--0.09 | 0.23 | 1.33 x10^-04^ | 0.11-0.35 |
|  | **MOCA** | 0.08 | 0.457 | -0.13-0.29 | -0.13 | 0.235 | -0.34-0.08 |
|  | **UPDRS III** | -1.49 | 6.45 x10^-04^ | -2.35--0.64 | 1.66 | 1.50 x10^-04^ | 0.80 - 2.51 |
